# 120 The Association of Admission Cultures with Burn Outcomes

**DOI:** 10.1093/jbcr/irac012.122

**Published:** 2022-03-23

**Authors:** Maree Baird, Joyce E Wall, Angela Man, Najib M Allabadi, Marc G Jeschke, Alisa Savetamal, John T Schulz, Kathleen S Skipton Romanowski

**Affiliations:** Shriners Hospitals for Dallas, Sacramento, Sacramento, California; Bridgeport Hospital, Seymour, Connecticut; Massachusetts General Hospital, Boston, Massachusetts; Vohra physcian group, Fresno, California; U of T, Toronto, Ontario; Yale New Haven System/Bridgeport Hospital, Bridgeport, Connecticut; Massachusetts General Hospital, Boston, Massachusetts; UC Davis, Sacramento, California

## Abstract

**Introduction:**

Burn patients are susceptible to infections. It is thought that burn wounds are initially sterile and become colonized by commensal and environmental microorganisms. Many burn centers have protocols to routinely screen patients for infection on admission. The ability of culture results to predict outcomes in burn patients has not been examined. In this study, we aim to examine the relationship between admission cultures and burn outcomes. We hypothesize that patients who have positive cultures on admission will have increased mortality and length of stay (LOS).

**Methods:**

A retrospective chart review was conducted using electronic medical records for all adult patients admitted to three ABA verified burn centers from January 2016 -December 2017. Data collected included patient demographics, burn injury, burn outcomes, and cultures obtained within the first 24 hours of admission. Data analysis was conducted using Chi-square, Fisher Exact, Spearman Correlation, Wilcoxon 2-sample, and Kruskal-Wallis tests.

**Results:**

A total of 1615 patients (mean age 45.87±17.65 years, 1145 males [70.9%]) were analyzed. Mean total body surface area burn (TBSA) was 9.6±14.2% and 10% had inhalation injuries. In this study population, the median LOS was 7 days (Interquartile range [IQR] = 12) and 72 patients (4.5%) expired. Older patients (*p* < .0001), those with higher scores on the 11-factor modified frailty index (mFI-11) (p < 0.0001), a higher TBSA (p< .0.0001) and inhalation injury (p < 0.0001) had a higher mortality rate. In examining the effect of admission cultures on mortality, there was no significant difference in mortality based on wound culture (*p* 0.14), *Clostridium difficile* (C. diff) (*p* 0.25), or urine culture (p=0.79) results. Patients with positive Methicillin-Resistant Staphylococcus Aureus (MRSA) screening (*p* 0.04) and those with positive blood cultures (*p* 0.01) were more likely to die from their injuries. Older patients (r= 0.14, *p* < 0.0001), those with a larger TBSA (r=0.49, *p* < 0.0001), and a higher MFI-11 score (r=0.12, *p* < 0.0001) had and increased LOS.. There was no association between LOS and positive wound cultures (*p* 0.08), or blood cultures (*p* 0.49) upon admission. Patients with positive MRSA results (*p* 0.003) and urine cultures (*p* 0.01) upon admission had a longer LOS while those with positive C. diff results had a shorter LOS (*p* 0.01).

**Conclusions:**

Mortality is associated with standard predictors of outcomes (age, burn size, inhalation injuries, frailty scores) and positive MRSA screens and blood cultures. Patients with larger burns (define larger burn-maybe use the degree scale), a positive MRSA and negative C. diff had a longer LOS. Based on these results, cultures should be considered in all patients upon admission to the hospital as they are predictive of burn outcomes.

